# The Prevalence and Independent Risk Factors of Significant Tricuspid Regurgitation Jets in Maintenance Hemodialysis Patients With ESRD

**DOI:** 10.3389/fphys.2020.568812

**Published:** 2020-12-17

**Authors:** Ying Zhang, Xiao-Han Ding, Fang Pang, Laiping Zhang, Yiqin Wang, Weili Wang, Rongsheng Rao, Shi-Zhu Bian

**Affiliations:** ^1^Department of Nephrology, The Key Laboratory for the Prevention and Treatment of Chronic Kidney Disease of Chongqing, Kidney Center of PLA, Xinqiao Hospital, Army Medical University (Third Military Medical University), Chongqing, China; ^2^Department of Health Care and Geriatrics, The 940th Hospital of PLA Joint Logistics Support Force, Lanzhou, China; ^3^Institute of Cardiovascular Diseases of PLA, Army Medical University (Third Military Medical University), Chongqing, China; ^4^Department of Cardiology, Xinqiao Hospital, Chongqing, China; ^5^Department of Ultrasound, Xinqiao Hospital, Army Medical University (Third Military Medical University), Chongqing, China

**Keywords:** prevalence, tricuspid regurgitation, maintenance dialysis, end-stage renal disease, retrospective study

## Abstract

**Background and Aim:**

Tricuspid regurgitation (TR) is a frequent complication in various cardiovascular diseases. However, few studies have reported the prevalence of TR especially the moderate to severe or significant TR (ms-TR) maintenance dialysis patients. Thus, we aimed to identify the prevalence of ms-TR and its associated factors.

**Methods:**

A total of 491 maintenance dialysis patients underwent echocardiographic examinations, while a subgroup (*n* = 283) also received routine blood tests, renal function examinations, and electrolyte analysis. We first compared the differences in abovementioned parameters among groups with various TR areas (TRAs). Finally, univariate and adjusted regression were also used to identify factors that were independently associated with ms-TR.

**Results:**

The incidence of TR jets was 62.6%, which included a mildly increased TRA (47.8%), moderately increased TRA (10.4%), and severely increased TRA (3.5%). Most of the cardiac structures and functional parameters, such as the end-diastolic internal diameters of the left atrium (LA), left ventricle (LVDD), right atrium (RA), right ventricle (RV), left ventricular ejection fraction (LVEF), and fractional shortening (FS), were significantly associated with ms-TR. Among serum ions, only total CO_2_ (TCO_2_; *r* = −0.141, *p* = 0.047) was negatively correlated with TRA. After adjusted, only Na^+^ [odds ratio (OR): 0.871 0.888, *p* = 0.048], RA (OR: 1.370, *p* < 0.001), and FS (OR: 0.887, *p* < 0.001) were independently associated with ms-TR.

**Conclusion:**

Tricuspid regurgitation occurs in maintenance hemodialysis patients with ESRD. Na^+^ FS and RA were independently associated with ms-TR, and these parameters may be potential risk factors/predictors for ms-TR.

## Introduction

End-stage renal disease (ESRD) has been demonstrated to be accompanied by various cardiovascular complications, which usually have poor outcomes (Kim and Parekh, 2015; Ramesh et al., 2016; Samanta et al., 2019). It has been reported that cardiovascular events, including heart failure, are the leading causes of death in ESRD patients (Kooman and van der Sande, 2019; Sugiura et al., 2019; Yogasundaram et al., 2019). The progressively deteriorating structure and function of the heart may be the consequence of renal failure (Cutshall et al., 2018; Unlu et al., 2018). On other hand, the insufficient perfusion of the kidney from the heart may aggravate renal dysfunction. Previous studies mainly focused on structural and functional cardiac changes in the left heart in ESRD patients and identified several biochemical markers, clinical factors, and hemodynamic predictors (Mahfouz et al., 2013; Minami et al., 2016; Lemor et al., 2018; Khan et al., 2019; Yogasundaram et al., 2019).

However, the structure and function of the right heart in ESRD patients, especially those who underwent dialysis treatment, have not been extensively studied. Tricuspid regurgitation (TR), which includes functional or mild TR, moderate TR and significant severe TR, is one of the most important functions of the right heart and is highly prevalent in various populations (Agricola et al., 2017; Al-Hijji et al., 2017; Sun and O’Gara, 2017). However, the prevalence and associated factors have not been identified in ESRD patients who received maintenance dialysis. TR may contribute to renal dysfunction in heart failure patients (Maeder et al., 2008).

Many previous studies have been studied the roles of tricuspid regurgitation in right heart failure in hemodialysis patients (Gumus and Saricaoglu, 2020). Part of the associated factors have also been found. However, interaction and relationship between TR especially ms-TR or significant TR and renal dysfunction attached less attentions. Furthermore, the prevalence of ms-TR in the special subgroup populations of dialysis patients has not been studied deeply (Cakici et al., 2020). Thus, we performed this retrospective study to identify the prevalence of ms-TR and potentially associated factors in a large sample size of a special population: ESRD patients who underwent maintenance dialysis.

## Materials and Methods

### Study Design and Populations

**FIGURE 1:**
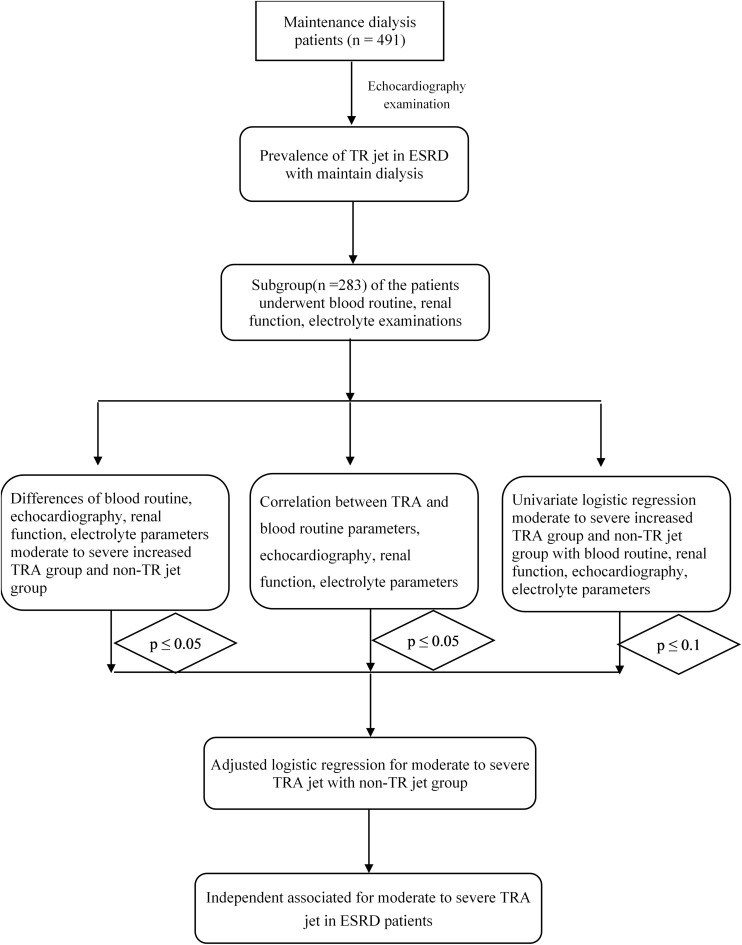
The flow chart of this study.

From March 2011 to June 2019, ESRD patients who received maintenance dialysis (*n* = 491) at the Nephrology of Chongqing and Kidney Center of PLA, Xinqiao Hospital, and Army Medical University (Third Military Medical University) were included in this retrospective study. These hemodialysis patients underwent echocardiographic examinations, while a subgroup (*n* = 283) of this population also received routine blood tests, renal function examinations, and electrolyte analysis, which are shown in Figure 1.

All of the ESRD patients were informed about the purpose and procedures of our study and provided written informed consent. Our present study complied with the Declaration of Helsinki.

In addition, the ethics committee of the Second Affiliated Clinic Hospital (Xinqiao Hospital) and Army Medical University (Third Military Medical University) approved the study.

### Procedures of Echocardiographic Examinations

The echocardiographic examinations of each patient were carried out by a skilled echocardiographer, Dr. Rongsheng Rao, according to the American Society of Echocardiography recommendations (Mansour et al., 2010). Echocardiographic examinations were performed in a supine position after resting for 10 min after hemodialysis. Two-dimensional echocardiographically guided motion mode (M-mode) images were obtained from standardized views by using an ultrasonography system (CX50, Philips, United States) with an S5 probe.

Among the echocardiographic parameters, our trained physicians recorded the left atrial end-diastolic internal diameter (LA), left ventricular end-diastolic internal diameter (LVDD), right atrial end-diastolic internal diameter (RA), right ventricular end-diastolic internal diameter (RV), and pulmonary arterial end-diastolic internal diameter (PA).

Additionally, the mobility and size of the left ventricular posterior wall (LVPW), the mobility and the size of the interventricular septum (IVS), fractional shortening (FS) and left ventricular ejection fraction (LVEF) and stroke volume (SV) were also recorded. Finally, TR-related parameters, such as TR area (TRA), TR velocity (TRV), and TR pressure, were also measured and recorded.

### Biomarker Determination

After admission to the hospital, we collected venous blood samples early in the morning after patients had fasted for more than 12 h before dialysis. The blood samples have also been obtained after dialysis in order to assess the effect of dialysis.

First, renal function, such as eGFR, plasma creatinine (Cr) and the blood urea nitrogen (BUN) concentration, was examined. Cr was also tested with Roche Diagnostics GmbH products (enzyme assay, Abbott, i2000, United States). BUN was also measured with ferene methods (Beckman AU5821, GA, United States).

Then, we performed routine blood examinations. An automated hematology chemistry analyzer was used to examine the hemoglobin concentration (Hb), red blood cell (RBC) count (RBC), hematocrit (HCT), mean corpuscular volume (MCV), mean corpuscular hemoglobin (MCH), mean corpuscular hemoglobin concentration (MCHC), and red blood cell distribution width (RDW) (type: AU400; Olympus Optical, Co., Tokyo, Japan). Furthermore, we also examined the white blood cell (WBC) and platelet (PLT) counts. The lymphocyte ratio (LYM), basophil ratio (BAS), neutrophil ratio (NEU), eosinophil ratio (EO), and monocyte ratio (MONO) were also tested and recorded.

Finally, we also performed an electrolyte examination, which included the sodium concentration (Na^+^, indirect ion selective electrode assay with EX-Z, JOKOH, Japan), serum calcium concentration (Ca^2+^, tri-azo methods), and phosphate concentration (P, phosphomolybdate ultraviolet assay, Roche Diagnostics GmbH, United States). In addition, we also measured the serum potassium concentration (K^+^, ferene methods, Beckman AU5821).

### Variable Definitions

First, patients were divided into the non-TR group (without a TR jet), the mildly increased TRA group (0 < TRA < 5 cm^2^) and the moderately to severely increased TRA group (ms-TR, TRA ≥ 5 cm^2^) which is also called significant TR (ms-TR in this study).

We calculated the estimated glomerular filtration rate (eGFR) by using the Chronic Kidney Disease Epidemiology Collaboration (CKD-EPI) formula: eGFR (ml/min/1.73 m^2^) = 1.41 × min(Cr/k,1)^α^ × max(Cr/,1)^–1.209^ × 0.993^*age*^ × 1.018 [*k* = 0.7 (female) and 0.9 (male); α = −0.329 (female) and −0.411 (male)].

### Statistical Analysis

If a continuous variable was normally distributed, it is presented as the mean ± standard deviation (SD). We employed one-way ANOVA to compare differences among the non-TR, mildly increased TRA and moderately to severely increased TRA groups. Further analysis comparing two groups was performed using least significant difference (LSD) methods after one-way ANOVA.

If one variable was non-normally distributed, it is presented as the median (25–75%). Comparisons among the non-TR, mildly increased TRA and moderately to severely increased TRA groups of variables with non-normal distributions were performed by one-way ANOVA after being transformed to achieve normality.

We performed univariate logistic regression analyses with each variable to screen for factors associated with moderate to severe TR. Variables with a *p* value less than 0.1 in the univariate logistic regression were included in multivariate (adjusted) logistic regression analyses to identify factors that were independently associated with moderate to severe TR. Our statistical analyses were performed by using the statistical software SPSS 22.0 (CA, United States) for Mac.

## Results

### Basic Information and the Prevalence of TR in the Study Populations

**FIGURE 2:**
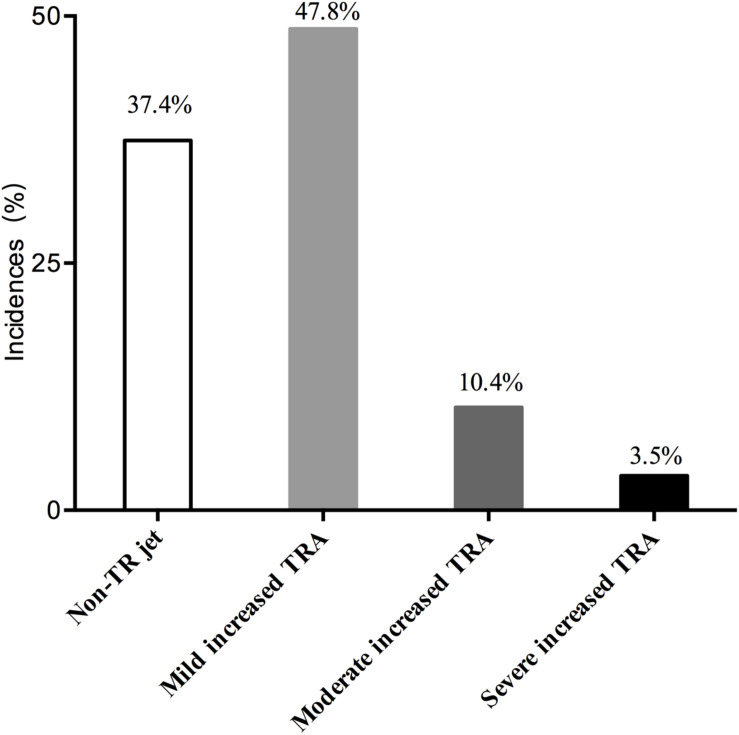
Distributions of TRA in the ESRD patients.

In our study, 491 ESRD patients were included. The mean age was 52.95 ± 14.21 years, and the mean body-mass index (BMI) was 21.89 ± 4.89 kg/m^2^. The incidence of a TR jet was 62.6%, which included a mildly increased TRA (47.8%), a moderately increased TRA (10.4%), and a severely increased TRA (3.5%). Thus, the incidence of ms-TR was 13.9%. These results are shown in Figure 2.

### Differences in Demographic, Echocardiographic, Routine Blood, Electrolyte and Renal Function Parameters Among Various TRA Groups

First, we did not find any differences in the demographic data (age and BMI, *p* > 0.05) among the non-TR, mildly increased TRA and ms-TR groups.

Second, in the echocardiographic parameters, both left heart and right heart structures and functions showed significant differences among these three groups. LA (43.93 ± 6.58 vs. 36.74 ± 4.75) and LVDD (54.11 ± 7.18 vs. 48.80 ± 5.25) were significantly higher in the ms-TR group than in the non-TR group (all *p* values < 0.001). These parameters were also significantly higher in the ms-TR group than in the mildly increased TRA group (all *p* values < 0.001). However, there were no differences in LA and LVDD between the mildly increased TRA and non-TR groups (all *p* values > 0.05). Though there were no differences in IVS and LVPW thickness among the three subpopulations, and their mobility was significantly different among the various TRA groups. ms-TR patients had worse IVS mobility [3.00 (6.00–6.00)] and a more dispersive LVPW mobility [10.00 (7.00–10.00)] than the other two groups (all *p* values < 0.001, Table 1). FS was also significantly lower in the ms-TR population [29.00 (18.75–33.25)] than in the non-TR [35.00 (32.00–37.00), *p* < 0.001] and mildly increased TRA [34.00 (32.00–37.00), *p* < 0.001] populations. The left heart functions, including LVEF, were significantly different among the three groups. The ms-TR group was characterized by a lower LVEF (50.28 ± 13.93%) than the other two subgroups (62.55 ± 8.01 and 62.28 ± 7.78, *p* < 0.001). However, SV was similar in the three groups (all *p* values > 0.05). In addition, PA was also significantly higher in the ms-TR group than in the other groups (25.14 ± 2.58 vs. 23.65 ± 2.44 and 23.68 ± 2.49 mm, *p* = 0.001, Table 1).

**TABLE 1:** Differences between of the hemodynamics, demographic data, renal function, electrolyte and routine blood examination parameters among various TRA groups.

	Overall	Non –TR patients	TRA increased groups		
			
Parameters	(*n* = 491 Subgroup *n*_1_ = 283)	(*n* = 84)	Mild increased TR (*n* = 153)	ms-TR group (*n* = 46)	*p* value
***Demographic data***					
Age (years)	52.54 ± 14.32	52.23 ± 12.50	53.22 ± 15.25	51.41 ± 14.19	0.719
BMI (kg/m^2^)	22.45 ± 3.56	22.54 ± 3.17	22.56 ± 3.79	21.87 ± 3.92	0.503
**Echocardiographic parameters**					
LA (mm)	38.41 ± 5.77	36.74 ± 4.75	38.02 ± 4.86	43.93 ± 6.58**^##^	<0.001
LVDD (mm)	49.30 ± 6.29	48.80 ± 5.25	48.92 ± 5.93	54.11 ± 7.18**^##^	<0.001
RA (mm)	37.15 ± 5.11	35.52 ± 3.78	36.43 ± 4.03	44.41 ± 5.36**^##^	<0.001
RV (mm)	35.46 ± 5.56	34.40 ± 3.40	34.90 ± 3.51	40.98 ± 5.03**^##^	<0.001
PA (mm)	23.82 ± 2.70	23.65 ± 2.44	23.68 ± 2.49	25.14 ± 2.58**^##^	0.001
IVS (mm)	13.04 ± 8.57	12.65 ± 1.64	12.47 ± 1.57	12.22 ± 1.73	0.345
IVS mobility	6.00 (6.00–6.00)	6.00 (6.00–6.00)	6.00 (6.00–6.00)	3.00 (6.00–6.00) **^##^	<0.001
LVPW (mm)	11.00 (10.00–12.00)	11.00 (10.00–12.00)	11.80 (10.00–12.00)*	11.20 (10.00–12.00)	0.144
LVPW mobility	10.00 (10.00–10.00)	10.00 (10.00–10.00)	10.00 (10.00–10.00)	10.00 (7.00–10.00)**^##^	<0.001
FS (%)	34.00 (31.00–37.00)	35.00 (32.00–37.00)	34.00 (32.00–37.00)	29.00 (18.75–33.25)**^##^	<0.001
LVEF (%)	60.83 ± 9.44	62.55 ± 8.01	62.28 ± 7.78	50.28 ± 13.93**^##^	<0.001
SV (ml)	75.10 ± 20.22	74.57 ± 15.53	78.41 ± 21.86	75.20 ± 18.43**^##^	0.301
TRV (cm/s)	281.00 (243.00–336.00)	–	265.00 (240.00–311.50)	354.00 (322.00–384.25)	<0.001
ΔP (mmHg)	33.00 (22.00–46.00)	–	28.00 (23.00–38.25)	50.00 (40.50–59.00)	<0.001
**Renal function (*n* = 283)**					
eGFR [mL/(min⋅1.73 m^2^)]	10.47 (5.62–13.00)	10.47 (5.88–12.04)	10.03 (5.88–13.00)	10.34 (5.28–13.50)	0.981
Cr (μmol/L)	815.50 (618.40–1016.90)	821.80 (618.92–1017.45)	814.40 (598.00–998.25)	817.35 (643.95–1126.22)	0.448
BUN (μmoI/L)	21.75 ± 7.82	21.26 ± 7.64	21.77 ± 7.58	22.57 ± 8.98	0.659
***Electrolyte parameters***					
Mg^2+^ (mmol/L)	0.982 ± 0.158	0.983 ± 0.175	0.978 ± 0.153	0.991 ± 0.143	0.877
K^+^ (mmol/L)	4.79 ± 0.787	4.78 ± 0.72	4.78 ± 0.79	4.83 ± 0.89	0.944
Ca^2+^ (mmol/L)	2.15 (2.03–2.28)	2.15 (2.05–2.280)	2.15 (2.02–2.28)	2.14 (1.97–2.36)	0.957
Na^+^(mmol/L)	137.85 (136.40–139.80)	138.40 (136.50–140.00)	137.84 (136.35–140.0)	137.35 (136.07–139.20)	0.149
Cl^–^ (mmol/L)	102.45 (99.60–105.30)	102.45 (99.10–105.20)	102.45 (99.60–105.50)	102.45 (99.98–105.80)	0.882
TCO_2_ (mmol/L)	20.66 (18.00–23.00)	21.05 (18.78–23.95)	20.50 (18.10–23.00)	19.90 (16.75–22.02)	0.075
P (mmol/L)	1.91 ± 0.64	1.96 ± 0.63	1.89 ± 0.62	1.91 ± 0.69	0.722
Dialysis time (days)	1230.00 (541.00–2549.00)	1214.00 (497.00–2101.25	1312.00 (539.00–2619.00)	1368.00 (555.750–2632.75)	0.183
**Routine blood examination parameters**					
RBC (10^12^/L)	3.39 ± 0.72	3.37 ± 0.62	3.40 ± 0.73	3.39 ± 0.86	0.950
Hb (g/dl)	100.52 ± 19.42	99.98 ± 19.46	100.45 ± 19.07	101.74 ± 20.83	0.884
Hct (L/L)	31.85 ± 6.12	31.85 ± 6.07	31.70 ± 5.98	32.37 ± 6.78	0.809
MCV (fl)	95.14 ± 8.02	95.11 ± 8.11	94.75 ± 8.14	96.50 ± 7.46	0.431
MCH (pg)	30.03 ± 2.70	29.84 ± 2.57	30.03 ± 2.78	30.39 ± 2.65	0.547
MCHC (g/dl)	315.72 ± 12.98	313.92 ± 11.22	316.99 ± 13.97	314.79 ± 12.38	0.191
RDW-CV (%)	14.57 ± 1.65	14.65 ± 1.61	14.48 ± 1.71	14.69 ± 1.56	0.649
RDW-SD (%)	51.30 (47.00–55.40)	51.84 (47.00–55.78)	51.20 (46.75–54.15)	51.47 (48.70–56.60)	0.342
PLT (10^9^/L)	167.00 (124.00–211.00)	171.756 (127.50–212.50)	165.00 (123.00–221.00)	150.00 (126.75–197.75)	0.258
WBC (10^9^/L)	6.61 (5.22–8.18)	6.44 (5.24–8.09)	6.87 (5.22–8.40)	6.07 (5.10–6.98)	0.445
EO (%)	2.80 (1.60–4.70)	2.65 (1.50–4.35)	2.70 (1.50–4.85)	3.35 (1.88–5.00)	0.277
BASO (%)	0.500 (0.300–0.700)	0.500 (0.200–0.700)	0.500 (0.300–0.700)	0.500 (0.200–0.800)	0.763
MONO (%)	6.00 (4.60–7.50)	6.05 (4.48–6.98)	5.90 (4.55–7.60)	6.38 (4.80–7.62)	0.771
NEU (%)	71.43 ± 9.54	70.77 ± 9.82	72.16 ± 9.36	70.19 ± 9.59	0.352
LYMP (%)	17.78 ± 7.23	18.20 ± 6.87	17.16 ± 7.05	19.03 ± 8.37	0.249

*p* presents differences among various groups.

*: p < 0.05 compared with non-TR group; **: *p* < 0.01 compared with non-TR group; ##: *p* < 0.01 compared with mild increased TR group.

In regard to renal function, none of the parameters (eGFR, Cr, and BUN) were significantly different among the three groups of patients (all *p* values were greater than 0.05, Table 1).

We further compared the differences in serum ions [Mg^2+^, K^+^, Ca^2+^, Na^+^, Cl^–^, total CO_2_ (TCO_2_), and P]. However, none of these ions were significantly different among the non-TR, mildly increased TRA and ms-TR groups (Table 1).

Finally, we also investigated routine blood examinations. However, we did not find any differences among the three groups (Table 1).

### Correlation Between TRA and Demographic, Echocardiographic, Routine Blood, Electrolyte and Renal Function Parameters

To identify associations between TRA and other parameters, we performed Spearman’s correlation analysis. We found that LA (*r* = 0.510, *p* < 0.001), LVDD (*r* = 0.389, *p* < 0.001), RA (*r* = 0.615, *p* < 0.001), RV (*r* = 0.586, *p* < 0.001), and PA (*r* = 0.322, *p* < 0.001) were closely positively related to TRA. Meanwhile, the IVS mobility (*r* = −0.338, *p* < 0.001), LVPW mobility (*r* = −0.344, *p* < 0.001), LVEF (*r* = −0.401, *p* < 0.001), and FS (*r* = −0.403, *p* < 0.001) as well as TCO_2_ (*r* = −0.141, *p* = 0.047) were negatively correlated with TRA. We did not find other associations between TRA and the rest of the parameters (Table 2).

**TABLE 2:** Relationship between TRA and other parameters.

	Relationship with TRA	
	
Parameters	*R*	*p* value
***Demographic data***		
Age (years)	–0.064	0.371
BMI (kg/m^2^)		
**Echocardiographic parameters**		
LA (mm)	0.510	<0.001
LVDD (mm)	0.389	<0.001
RA (mm)	0.615	<0.001
RV (mm)	0.586	<0.001
PA (mm)	0.322	<0.001
IVS (mm)	0.086	0.228
IVS mobility	–0.338	<0.001
LVPW (mm)	0.084	0.237
LVPW mobility	–0.344	<0.001
FS (%)	–0.403	<0.001
LVEF (%)	–0.401	<0.001
SV (ml)	0.095	0.184
**Renal function**		
eGFR [mL/(min⋅1.73 m^2^)]	0.047	0.510
Cr (umol/L)	0.013	0.858
BUN (μmoI/L)	0.056	0.433
**Electrolyte parameters**		
Mg^2+^ (mmol/L)	0.080	0.263
K^+^ (mmol/L)	0.088	0.215
Ca^2+^ (mmol/L)	–0.039	0.583
Na^+^(mmol/L)	–0.012	0.867
Cl^–^ (mmol/L)	0.109	0.127
TCO_2_ (mmol/L)	–0.141	0.047
P (mmol/L)	0.067	0.345
Dialysis time (days)	–0.046	0.518
**Blood routine examination parameters**		
RBC (10^12^/L)	0.033	0.641
Hb (g/dl)	0.062	0.381
Hct (L/L)	0.075	0.292
MCV (fl)	0.063	0.376
MCH (pg)	0.043	0.546
MCHC (g/dl)	–0.027	0.709
RDW-CV (%)	0.021	0.765
RDW-SD (%)	0.060	0.402
PLT (10^9^/L)	–0.080	0.260
WBC (10^9^/L)	–0.114	0.109
EO (%)	–0.054	0.449
BASO (%)	0.041	0.563
MONO (%)	0.058	0.418
NEU (%)	–0.023	0.750

### Logistic Regressions for ms-TR With Demographic, Echocardiographic, Routine Blood, Electrolyte and Renal Function Parameters

In univariate regression analysis, demographic data (age and BMI) were not associated with ms-TR (all *p* values > 0.05) (Table 3).

**TABLE 3:** Univariate logistic analysis for ms-TR jet.

				95CI%	
				
Parameters	β	*p* value	OR	Lower borderline	Upper borderline
***Demographic data***					
Age (years)	–0.005	0.734	0.995	0.968	1.023
BMI (kg/m^2^)	–0.057	0.291	0.945	0.850	1.050
Echocardiographic parameters					
LA (mm)	0.248	<0.001	1.281	1.166	1.408
LVDD (mm)	0.139	<0.001	1.149	1.076	1.227
RA (mm)	0.362	<0.001	1.437	1.281	1.612
RV (mm)	0.343	<0.001	1.410	1.251	1.589
PA (mm)	0.233	0.003	1.262	1.083	1.471
IVS (mm)	–0.162	0.163	0.850	0.677	1.068
IVS mobility	–0.821	<0.001	0.440	0.295	0.655
LVPW (mm)	0.058	0.559	1.060	0.872	1.288
LVPW mobility	–0.531	<0.001	0.588	0.449	0.770
FS (%)	–0.159	<0.001	0.853	0.801	0.909
LVEF (%)	–0.100	<0.001	0.905	0.869	0.943
SV (ml)	0.002	0.835	1.002	0.981	1.024
Dialysis time (days)	0.001	0.203	1.000	1.000	1.001
**Renal function**					
eGFR [mL/(min⋅1.73 m^2^)]	0.023	0.374	1.023	0.973	1.075
Cr (umol/L)	0.001	0.456	1.000	0.999	1.001
BUN (μmoI/L)	0.020	0.380	1.020	0.976	1.066
Electrolyte parameters					
Mg^2+^ (mmol/L)	0.029	0.796	1.337	0.149	12.006
K^+^ (mmol/L)	0.077	0.744	1.080	0.682	1.709
Ca^2+^ (mmol/L)	0.118	0.861	1.125	0.300	4.218
Na^+^(mmol/L)	–0.146	0.044	0.864	0.749	0.996
Cl^–^ (mmol/L)	0.022	0.608	1.022	0.939	1.113
TCO_2_ (mmol/L)	–0.116	0.017	0.890	0.809	0.980
P (mmol/L)	–0.105	0.711	0.901	0.517	1.568
**Routine blood examination parameters**					
RBC (10^12^/L)	0.057	0.827	1.058	0.637	1.759
Hb (g/dl)	0.005	0.628	1.005	0.986	1.023
Hct (L/L)	0.013	0.655	1.013	0.957	1.073
MCV (fl)	0.023	0.339	1.023	0.976	1.072
MCH (pg)	0.084	0.255	1.088	0.941	1.258
MCHC (g/dl)	0.007	0.681	1.007	0.976	1.039
RDW-CV (%)	0.014	0.903	1.014	0.808	1.273
RDW-SD (%)	0.026	0.336	1.026	0.974	1.081
PLT (10^9^/L)	–0.003	0.242	0.997	0.991	1.002
WBC (10^9^/L)	–0.032	0.710	0.969	0.820	1.145
EO (%)	–0.006	0.742	0.994	0.958	1.031
BASO (%)	–0.025	0.544	0.976	0.901	1.057
MONO (%)	0.030	0.951	1.030	0.404	2.625
NEU (%)	0.015	0.542	1.015	0.967	1.066
RBC (10^12^/L)	0.063	0.441	1.065	0.907	1.252

*Primary screen for the independently associated factors.*

Among the echocardiographic parameters, we identified several factors that were potentially associated with ms-TR. First, the diameter of the left and right heart as well as the PA, LA [odds ratio (OR): 1.281, 95% confidence interval (CI): 1.166–1.408, *p* < 0.001], LVDD (OR: 1.149, 95% CI: 1.076–1.227, *p* < 0.001), RA (OR: 1.437, 95% CI: 1.281–1.612, *p* < 0.001), RV (OR: 1.410, 95% CI: 1.251–1.589, *p* < 0.001), and PA (OR: 1.262, 95% CI: 1.083–1.471, *p* = 0.003) were associated with ms-TR (Table 3).

Furthermore, it was also found that IVS mobility (OR: 0.440, 95% CI: 0.295–0.655, *p* < 0.001), LVPW mobility (OR: 0.588, 95% CI: 0.449–0.770, *p* < 0.001), FS (OR: 0.853, 95% CI: 0.801–0.909, *p* < 0.001), and LVEF (OR: 0.905, 95% CI: 0.869–0.943, *p* < 0.001) may also have been associated with ms-TR (Table 3).

However, none of the renal function parameters showed significant associations with ms-TR (Cr, BUN, and eGFR, Table 3).

Among the serum ions, we found that only Na^+^ (OR: 0.864, 95% CI: 0.749–0.996, *p* = 0.044) and TCO_2_ (OR: 0.890, 95% CI: 0.809–0.980, *p* = 0.017) were potentially related to ms-TR (Table 3).

Finally, in the routine blood examination, RBC- and WBC-related parameters showed no potential associations with ms-TR. In addition, the other parameters, such as the ratios of monocytes, lymphocytes, eosinophils, and basophilic granulocytes, were not associated with ms-TR (Table 3).

To identify factors that were independently associated with ms-TR, we performed an adjusted (multivariate) logistic regression. We found that only Na^+^ (OR: 0.871 95% CI: 0.760–0.999, *p* = 0.048), RA (OR: 1.370, 95% CI: 1.234–1.520, *p* < 0.001), and FS (OR: 0.887, 95% CI: 0.822–0.957, *p* < 0.001) were independently associated with ms-TR (Table 4).

**TABLE 4:** Adjusted logistic regressions for ms-TR.

				95%CI	
				
Parameters	β	*p* value	OR	Lower borderline	Upper borderline
RA (mm)	0.3150	<0.001	1.370	1.234	1.520
FS (%)	−0.120	<0.001	0.887	0.822	0.957
Na^+^ (mmol/L)	−0.137	0.048	0.871	0.760	0.999

*Na^+^, RA, and FS have been identified to be independently associated with ms-TR.*

## Discussion

In our present study, we found that TR was prevalent (62.6%) in 491 ESRD patients who received maintenance dialysis treatment. Though TCO_2_ and other renal functions were potentially associated with TR, adjusted regression showed that only Na^+^, FS, and RA were independently associated with ms-TR; thus, further cohort studies are warranted to confirm their causality.

### TR Was Prevalent in ESRD Patients Who Received Maintenance Dialysis Treatment

As discussed above, though TR is common in normal populations or populations with other cardiovascular diseases (Arsalan et al., 2017), TR was more prevalent and had a relatively higher incidence in a special subgroup, maintenance dialysis patients with ESRD (Walsh et al., 1998; Maeder et al., 2008; Al-Hijji et al., 2017; Del Forno et al., 2018; Dietz et al., 2019). Furthermore, we also found that ms-TR showed a higher incidence than other degrees of TRA. In particular, a few individuals had a TRA that was larger than 15 cm^2^; these patients needed further examination or treatment by the cardiovascular disease department. In the past, few studies had investigated the roles of TR, as well as the function and structure of the left heart, in renal function in heart failure patients (Minami et al., 2016; Cutshall et al., 2018; Konigsbrugge and Ay, 2019). However, no papers have reported the prevalence of TR in a large population of maintenance dialysis patients with ESRD. Our study was the first to describe the prevalence and TRA degree in patients who received dialysis at our center. Our data revealed that a TR jet occurred in maintenance dialysis patients with ESRD. Some patients had a TR jet with an extremely large area and may have needed cardiac surgery interventions. Thus, echocardiographic examinations are a highly valuable non-invasive method to evaluate the cardiovascular complications of ESRD, providing guidelines for the treatment of patients.

### TR Was Not Closely Associated With Renal Function in ESRD Patients Undergoing Hemodialysis

It has been indicated that kidney dysfunction may contribute to cardiovascular complications (Yogasundaram et al., 2019). We investigated renal function in ESRD patients, but the eGFR, Cr and BUN were not significantly different among the various TRA groups and were not associated with TRA. Though few previous studies have indicated that renal dysfunction affects cardiac structure and function (Minami et al., 2016; Cutshall et al., 2018; Lemor et al., 2018), we did not find a potential association of eGFR, BUN and Cr with ms-TR. Thus, these parameters were not included in the final adjusted logistic regression. Though our present study did not find an association of renal functions with ms-TR, the causal relationship between renal function and TR jets may need further cohort studies or basic studies to be confirmed.

### TR Was Significantly Associated With Serum Ions

Ions are known to play numerous key roles in signal transduction, protein synthesis, cell proliferation and differentiation. In ESRD patients, serum ion disturbances are the most common clinical manifestations. Thus, we also investigated the associations between serum ions (Mg^2+^, Ca^2+^, K^+^, Cl^–^, TCO_2_, Na^+^, and P) and TR jets. First, there were no significant differences in the abovementioned ions in the various TRA groups. Then, we found that TCO_2_ was closely negatively correlated with TRA. In the past several decades, researchers have found that TCO_2_ levels changed in uremia, indicating that renal dysfunction may alter TCO_2_ levels, resulting in changes in respiration and oxygen usage. If this situation is persistent, it may cause hypoxia-induced cardiac injury or changes in cardiac structure and function. Through further univariate regression analysis, we found that TCO_2_ and Na^+^ were potentially associated with an ms-TR jet. However, in the adjusted regression analysis, these factors were excluded by other variables and did not show independent associations with ms-TR.

### TR Showed No Close Relationship With Routine Blood Parameters

Anemia, which is the consequence of the insufficient production of EPO and a higher demand for EPO, is another critical complication of ESRD and leads to insufficient Hb and RBC production (Santoro and Canova, 2005; van der Meer et al., 2008; Yokoro et al., 2017). It proposed that the anemia may contribute to TR jet. Thus, we investigated the association between RBC-related parameters and WBC-related parameters. However, we did not find any associations between RBC/WBC-related parameters and TR. This result may have been caused by the effect of anemia on cardiovascular diseases; this may involve a long-term process that requires cohort studies to identify their associations.

### Na^+^, FS, and RA May Contribute to the Development of ms-TR

Our present study found that Na^+^ FS and RA were independently associated with ms-TR. The structure and function of the left heart showed an association with ms-TR, which may have been caused by the interactions between the left and right heart. Furthermore, changes in the right heart may result in relative or absolute tricuspid insufficiency, which may aggravate changes in structure and function (enlargement and right heart failure) (Al-Hijji et al., 2017; Sun and O’Gara, 2017; Hahn, 2019).

We have identified that Na^+^ was independently associated with TR which was in concordant with the previous studies that Na^+^ was correlated to the cardiovascular diseases in ERSD dialysis patients. This may be caused by the roles of Na+ in the vascular systems. In according to the previous studies, cardiovascular diseases are the common complications in ESRD patients especially in the maintenance dialysis subset populations (Cakici et al., 2020; Gumus and Saricaoglu, 2020). The precise mechanisms of Na^+^ in TR have not been widely investigated.

Our present results were consistent with previous studies on various cardiovascular diseases that are induced by a TR jet (Lee et al., 2010; Al-Hijji et al., 2017; Kopic et al., 2017; Mangieri et al., 2017). However, the roles of FS in TR jets have not been reported before. We found that FS was significantly associated with ms-TR, which was a novel finding that revealed that left heart functions may affect the right heart (due to preloading or other hemodynamic changes), which is partly in according with the previous studies (Badano et al., 2019; Dietz et al., 2019; Prihadi et al., 2019). However, the precise cause and mechanisms were not confirmed; thus, further cohort studies and research to uncover the basic mechanisms are warranted.

### Limitations

This was a retrospective study focusing on the prevalence of TR and its associated factors. However, there are several limitations that need to be improved in our future studies. First, our present study was a retrospective study that identified only the factors associated with ms-TR. The causality between clinical factors and ms-TR needs to be analyzed by further cohort studies. Another limitation is that we investigated only a few of the frequently used clinical parameters; however, there may be other valuable factors that should be examined. Finally, the parameters after dialysis were not included in this study, but these factors should be included in future research.

## Conclusion

Tricuspid regurgitation is also prevalent in maintenance hemodialysis patients with ESRD. Na^+^, FS, and RA were independently associated with ms-TR; these may be potential risk factors/predictors for ms-TR and warrant further cohort studies.

## Data Availability Statement

The raw data supporting the conclusions of this article will be made available by the authors, without undue reservation.

## Ethics Statement

The studies involving human participants were reviewed and approved by the Second Affiliated Clinic Hospital (Xinqiao Hospital) and Army Medical University (Third Military Medical University). The patients/participants provided their written informed consent to participate in this study.

## Author Contributions

S-ZB and YZ designed the study, drafted the manuscript, and performed the statistical analysis. S-ZB and X-HD critically reviewed and revised the manuscript for important intellectual content. RR performed the echocardiographic examinations and LZ recorded the measurements of TR-related parameters. YZ and X-HD collected the demographic data and performed routine blood examinations. YW, FP, and LZ performed the laboratory measurements. YW and WW obtained the dialysis-related data. All authors contributed to the article and approved the submitted version.

## Conflict of Interest

The authors declare that the research was conducted in the absence of any commercial or financial relationships that could be construed as a potential conflict of interest.

## Abbreviations

BAS: basophil ratio
BUN: blood urea nitrogen concentration
Ca^2+^: serum calcium concentration
Cr: plasma creatinine
eGFR: estimated glomerular filtration rate
ESRD: end-stage renal disease
EO: eosinophil ratio
FS: fractional shortening
Hb: hemoglobin concentration
HCT: hematocrit
IVS: interventricular septum
K^+^: serum potassium concentration
LA: left atrial end-diastolic internal diameter
LVDD: left ventricular end-diastolic internal diameter
LVEF: left ventricular ejection fraction
LVPW: left ventricular posterior wall
LYM: lymphocyte ratio
MCH: mean corpuscular hemoglobin
MCHC: mean corpuscular hemoglobin concentration
MCV: mean corpuscular volume
MONO: monocyte ratio
Na^+^: sodium concentration
NEU: neutrophil ratio
ms-TR: moderate to severe TRA
P: phosphate concentration
PA: pulmonary arterial end-diastolic internal diameter
PLT: platelet
RA: right atrial end-diastolic internal diameter
RBC: red blood cell count
RDW: red blood cell distribution width
RV: right ventricular end-diastolic internal diameter
SV: stroke volume
TR: tricuspid regurgitation
TRA: TR area
TRV: TR velocity
WBC: white blood cell count
Δ P: mitral transmitral pressure gradient.
